# Osteomicrobiology: A New Cross-Disciplinary Research Field

**DOI:** 10.1007/s00223-017-0336-6

**Published:** 2017-10-27

**Authors:** Claes Ohlsson, Klara Sjögren

**Affiliations:** Centre for Bone and Arthritis Research, Institute of Medicine, Sahlgrenska Academy, University of Gothenburg, Vita Stråket 11, 413 45 Gothenburg, Sweden

**Keywords:** Bone mass, Osteoporosis, Probiotics, Gut microbiota, Osteomicrobiology

## Abstract

The mutualistic interaction between the gut microbiota (GM) and its host profoundly shapes many aspects of our physiology. The composition and activity of the gut microbiota is modulated by environmental factors such as dietary habits and antibiotic treatments. In rodents, studies demonstrate that the GM is a crucial regulator of bone metabolism and that modulation of the GM composition by probiotic interventions can prevent castration-induced bone loss. Short-term colonization of germ-free mice with GM results in an activation of CD4+T cells, resulting in increased levels of pro-inflammatory cytokines in bone and thereby activation of osteoclastic bone resorption. Besides these immune-mediated effects on bone mass, the GM is involved in nutritional uptake and may, thereby, regulate overall body growth and bone sizes possibly mediated via altered IGF-I levels. We recently introduced a new term “osteomicrobiology” for the rapidly emerging research field of the role of the microbiota in bone health. This research field is aimed to bridge the gaps between bone physiology, gastroenterology, immunology, and microbiology. Future studies will determine if the GM is a novel therapeutic target for osteoporosis and if the GM composition might be used as a biomarker for fracture prediction.

## Background

Osteoporosis-related fractures are a huge economic burden on health care systems and fractures are associated with significant mortality and morbidity. The lifetime risk of any osteoporotic fracture is high in the western world and about 50% of women and 20% of men older than 50 years will have a fragility fracture in their remaining lifetime [1]. Osteoporosis is characterized by enhanced skeletal fragility due to reduction in bone quantity and/or quality. The risk of osteoporosis depends both on how much bone is acquired until peak bone mass is reached at 20–30 years of age and on the rate of the subsequent age-dependent bone loss. Twin and family studies have shown that between 50 and 85% of the variance in peak bone mass is genetically determined, but for age-related bone loss, environmental factors seem to play a more pronounced role [2].

The skeleton is remodeled by bone-forming osteoblasts (OBs) and bone-resorbing osteoclasts (OCLs) [3]. The skeleton provides structural support and serves as a niche for mesenchymal and hematopoietic progenitors. OBs are derived from pluripotent mesenchymal stromal cells, while OCLs are derived from hematopoietic stem cells that also generate immune cells [4]. OCLs are specifically derived from the myeloid–monocyte lineage of hematopoietic cells. The local microenvironment determines whether the myeloid precursor cell will differentiate into a macrophage, a myeloid dendritic cell, or an OCL. The presence of macrophage colony stimulating factor (M-CSF) leads to increased proliferation and survival as well as upregulated expression of receptor activator of nuclear factor-κB (RANK) in OCL precursor cells. This allows RANK ligand (RANKL) to bind and start the signaling cascade that leads to OCL formation [4].

Osteoimmunology is a field describing the interactions between the immune and the skeletal systems [5]. The association between inflammation and bone loss is well established, and in autoimmune diseases, such as rheumatoid arthritis, osteoclastic bone resorption is driven by inflammatory cytokines produced by activated T cells [6]. The estrogen deficiency at menopause results in increased formation and prolonged survival of osteoclasts. This is due to the loss of the immunosuppressive effects of estrogen, resulting in increased production of cytokines promoting osteoclastogenesis and direct effects of estrogen on OCLs [7, 8]. Studies indicate that low-grade inflammation affects physiological bone turnover and plays a role in pathological skeletal conditions such as osteoporosis. Moderately elevated serum levels of high-sensitivity C-reactive protein (hsCRP), as an estimate of low-grade systemic inflammation, are associated with low BMD, elevated bone resorption, BMD loss, and increased fracture risk [9–12]. Furthermore, blockade of the inflammatory cytokines, tumor necrosis factor alpha (TNFα), and interleukin 1 (IL-1), leads to a decrease in bone resorption markers in early postmenopausal women [13].

In recent years, the development of effective sequencing technologies has made it possible to study the composition of the GM and its importance for both health and disease. The GM constitutes trillions of microorganisms, mostly anaerobic bacteria, which collectively contain 150-fold more genes than our human genome. It is acquired at birth and can be considered a multicellular organ that communicates with and affects its host in numerous ways [14]. The composition of the GM is modulated by environmental factors such as diet and antibiotic treatments [15–17]. Bacterial components and molecules produced by the gut bacteria are known to affect endocrine cells in the gut, the enteric nervous system, gut permeability, and the immune system [18]. At homeostasis, the GM provides colonization resistance with epithelial and immune balance, protecting the host from invading pathogens. Perturbations in this balance can be caused by pathogens, antibiotic treatment, diet causing inflammation, tissue destruction, and dysbiosis that may lead to disease development [19]. Perturbed microbial composition has been postulated to be involved in a range of inflammatory conditions, within and outside the gut, including rheumatoid arthritis, multiple sclerosis, inflammatory bowel diseases, diabetes, food allergies, eczema, and asthma as well as obesity and the metabolic syndrome [20, 21]. Gut-associated inflammatory and autoimmune conditions have been associated with low bone mass, suggesting a connection between the gut and bone [22, 23].

## Effects of GM on Bone Metabolism

The use of gnotobiotic animals has made it possible to characterize the effects of the GM. Germ-free (GF) animals have immature mucosal immune systems with poorly developed gut-associated lymphoid tissue (GALT). Furthermore, GF mice have reduced number of CD4+T cells in the spleen and fewer and smaller germinal centers within the spleen, suggesting that the GM is capable of shaping systemic immunity [24]. Besides these drawbacks, studies have shown that gnotobiotic mouse models are useful to analyze the host–microbiota interactions [25].

**Fig. 1:**
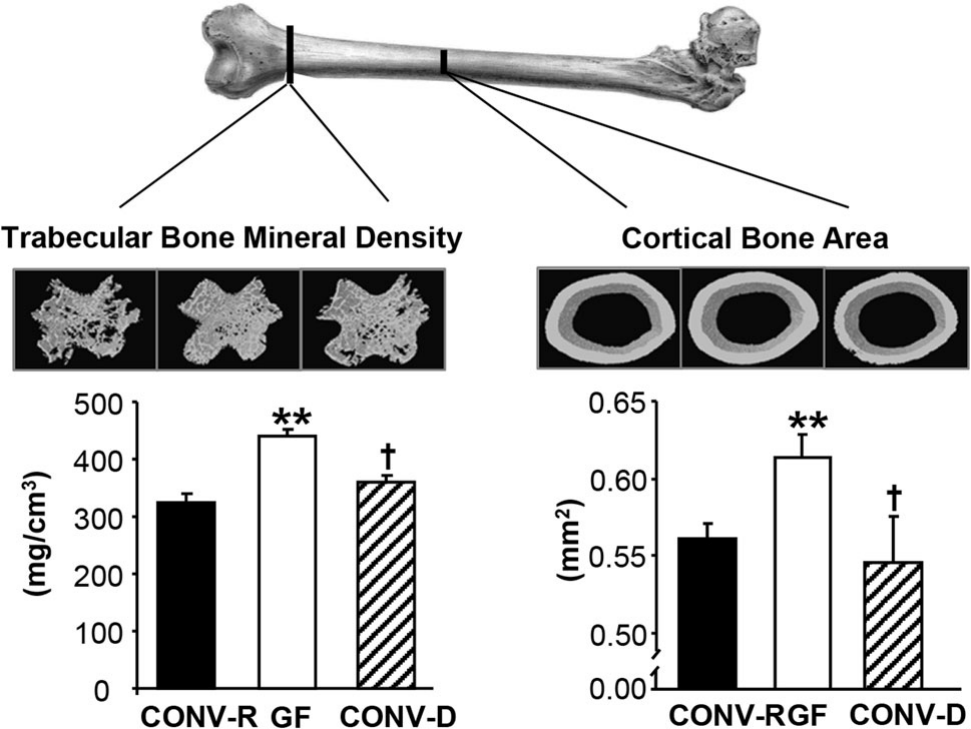
Absence of gut microbiota leads to increased bone mass in mice. Trabecular and cortical bone were analyzed by computed tomography in femur from germ-free (GF) and conventionally raised (CONV-R) female mice and an extra control group consisting of female mice that were born GF and then colonized with normal gut microbiota (conventionalized; CONV-D). Values are given as mean  ±  SEM, **p ≤ 0.01, GF versus CONV-R; ^†^ *p* ≤ 0.05, ^††^ *p* ≤ 0.01, CONV-D versus GF, ANOVA followed by Tukey’s post hoc test. Adapted from Sjogren et al. [26] with permission from the American Society for Bone and Mineral Research©

**Fig. 2:**
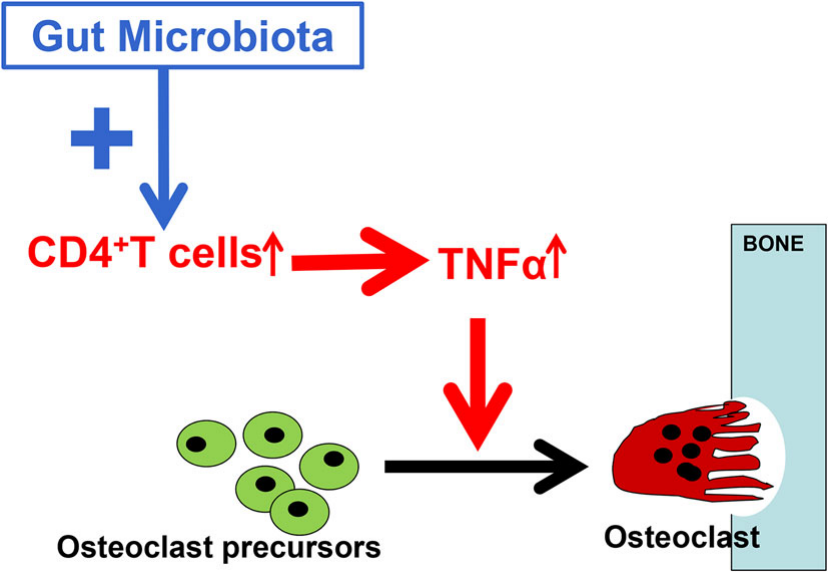
Immune-mediated effects of GM on bone metabolism. Colonization with GM leads to an activation of the immune system and specifically CD4+T cells, resulting in increased levels of pro-inflammatory cytokines in bone and bone marrow and thereby activation of osteoclastic bone resorption

Our group was the first to show that the absence of GM in GF mice leads to increased bone mass, compared to conventionally raised (CONV-R) mice [26]. We found that both the trabecular bone mass and the cortical bone mass were increased in the femur of GF compared with CONV-R mice [26]. The increased trabecular bone volume fraction was the result of increased trabecular number and decreased trabecular separation compared with CONV-R mice. In addition, cortical bone area was increased in GF mice (Fig. 1). To exclude confounding developmental effects, we colonized mice born in a GF environment with GM from mice raised in a conventional environment, demonstrating that both the trabecular and cortical bone mass were normalized (Fig. 1). GF mice had reduced number of osteoclasts per trabecular bone perimeter and a decreased number of OCLs were formed in bone marrow cultures from GF mice. In contrast, the bone formation rate was not significantly altered in the GF mice, suggesting that the increased bone mass in the GF mice was predominantly caused by reduced bone resorption, as a result of inhibited osteoclastogenesis. The GF mice had reduced mRNA levels of the osteolytic cytokines IL-6 and TNFα in the bone, strongly suggesting that the decreased osteoclastogenesis was caused by immune-mediated mechanisms. However, it cannot be excluded that other mechanisms might also contribute to the high bone mass phenotype we observed in GF mice. It is unlikely that the increased bone mass was caused by an altered calcium metabolism, because serum calcium and hormones regulating calcium homeostasis were normal in GF mice [26]. It has been reported, in some but not all studies, that gut-derived circulating serotonin inhibits bone formation and reduces bone mass [27, 28]. In our study, serum serotonin levels were decreased in GF mice; however, colonization of GF mice with GM from mice raised in a conventional environment, which leads to the normalization of bone mass, did not significantly alter serum serotonin levels, indicating that the high bone mass in GF mice most likely was not caused by altered serum serotonin levels [26]. After our study in 2012, there have been some other studies evaluating the bone mass in different GF mouse models. We observed increased bone mass but normal body growth and longitudinal bone growth in female C57BL/6 J mice and very similar results were observed by Li et al., also using female C57BL/6 J mice [29]. In a recent study by our group to determine the possible role of different innate immune signaling pathways for the effect of the GM on bone mass, we used 9-week-old female C57BL/6 J mice from the gnotobiotic facility at the Pasteur Institute, France [30]. Similar to our earlier studies using mice from the gnotobiotic facility at the University of Gothenburg, Sweden, we found that WT mice raised GF in the Pasteur Institute had increased bone mass of the femur diaphysis compared to CONV-R mice. Taken together, we, as well as others, have shown that GF mice have increased bone mass compared to CONV-R mice. In a recent study in male mice on a C57BL/6 background, GF mice exhibited increased trabecular bone mass, normal bone length, decreased osteoclast size, and eroded bone perimeter, which is similar to what we and Li et al. found using female mice on the same genetic background [26, 29, 31]. To summarize the above studies, colonization with GM leads to an activation of the immune system and specifically CD4 + T cells, resulting in increased levels of pro-inflammatory cytokines in bone and bone marrow and thereby activation of osteoclastic bone resorption (Fig. 2).

To determine the possible role of different innate immune signaling pathways for the effect of the GM on bone mass, we recently evaluated the skeleton in GF and CONV-R mice with targeted inactivation of Myd88, Nod1, or Nod2 [30]. We found that in contrast to WT and Myd88^−/−^ mice, cortical bone thickness in mice lacking Nod1 or Nod2 was not increased under GF conditions. The expression of TNFα and the osteoclastogenic factor RANKL in bone was reduced in GF compared to CONV-R WT mice but not in Nod1^−/−^ or Nod2^−/−^ mice indicating that the effect of the GM to increase TNFα and RANKL in bone and to reduce bone mass is dependent on both NOD1 and NOD2 signaling. It was recently demonstrated that muramyl dipeptide (MDP), known as a shared structural unit of peptidoglycans from both gram-positive and gram-negative bacteria and a ligand to NOD2, regulates bone mass indicating that the NOD2 signaling pathway is involved in GM effects on bone [32].

## Effects of GM on Body Growth and IGF-I

Body growth and the associated expansion of bone dimensions vary widely as a function of nutritional inputs and are regulated by the growth hormone (GH)/IGF-I axis. To maximize body growth and peak bone mass, balanced nutrition must be optimal before the onset of puberty and maintained throughout this period of rapid body growth [33]. Schwarzer et al. recently observed that 7-week-old male GF mice on a BALB/c background had a general growth defect reflected by reduced body weight gain as well as decreased longitudinal and radial bone growth compared to CONV-R mice [34]. The reduced overall body growth was associated with GH resistance and reduced IGF-I levels and the phenotype could be normalized by treatment with a specific microbial strain. These results suggest that the GM is required for optimal energy extraction from food and that the GM increases nutritional uptake and energy harvest and thereby may increase body growth depending on the given diet, the GM composition, and the genetic background of the mouse strain. In contrast, Novince et al. recently showed that male GF mice on a C57BL/6 background had increased serum IGF-I and normal body growth and femur length at 12 weeks of age [31]. We have observed normal bone length in female GF mice on a C57BL/6 J background in the gnotobiotic facility at the University of Gothenburg (own unpublished data). Differences in genetic background, diet, animal facility, sex, and GM composition may explain the conflicting results regarding the role of the GM on IGF-I levels and body growth.

A possible explanation could be that the growth-modulating effect of GM is crucial for body growth, bone dimensions and peak bone mass under certain conditions, while the more acute, immune-mediated effects of the GM on the skeleton is reflecting situations of adult bone metabolism such as postmenopausal osteoporosis and inflammation-mediated bone loss. In line with this, Yan et al. recently found that the short-term effect of the GM one month after colonization of GF mice was a reduction of bone mass, while long-term colonization increased overall body growth, resulting in enhanced longitudinal and radial bone growth [35]. The mice in this study were F1 hybrid CB6F1 made from crossing BALB/c with C57BL/6 mice. Short-term colonization resulted in a reduction of trabecular bone volume fraction and increased bone resorption indicated by increased serum CTX. This was also associated with increased levels of RANKL and TNFα in the gut and in bone marrow. Thus, for short-term colonization, GM induces the expression of inflammatory cytokines in bone, resulting in increased bone resorption and reduced bone mass very similar to that shown by us and others [26, 31, 35, 36]. However, the results from the long-term colonization evaluated 8 months after colonization were more similar to the growth phenotype observed by Schwarzer et al. The long-term colonization resulted in increased body growth associated with the expected increase in longitudinal and radial bone growth and increased serum IGF-I levels. To determine the mechanism for the effect of the GM on serum IGF-I, they then performed an experiment using antibiotic-treated mice. They observed that a reduction of the GM by antibiotic treatment resulted in a reduction of serum IGF-I and this could be reversed by treatment with short-chain fatty acids (SCFA) which is normally produced by the GM in the colon. Based on these data, they proposed that the GM results in fermentation of fibers in colon which results in increased levels of SCFA in the colon which by unknown mechanism increases IGF-I which in turn results in a general body growth associated with increased bone dimensions. In conclusion, the overall immune-related effect of short-term colonization of GF mice with GM is an activation of CD4+T cells, resulting in increased levels of pro-inflammatory cytokines in bone and thereby activation of osteoclastic bone resorption of importance for adult bone metabolism. Besides these immune-mediated effects on bone mass, the GM is involved in nutritional uptake and may, thereby, regulate overall body growth and bone sizes possibly mediated via altered IGF-I levels.

## Treatment with Probiotics and Effects on Bone

Probiotics is defined as live commensal microorganisms such as bacteria or fungi that when administered in adequate amounts can confer a health benefit on the host such as improved intestinal function and integrity of the intestinal lining. They may also positively affect immune responses in the host gastrointestinal tract to promote health [37].

**Fig. 3:**
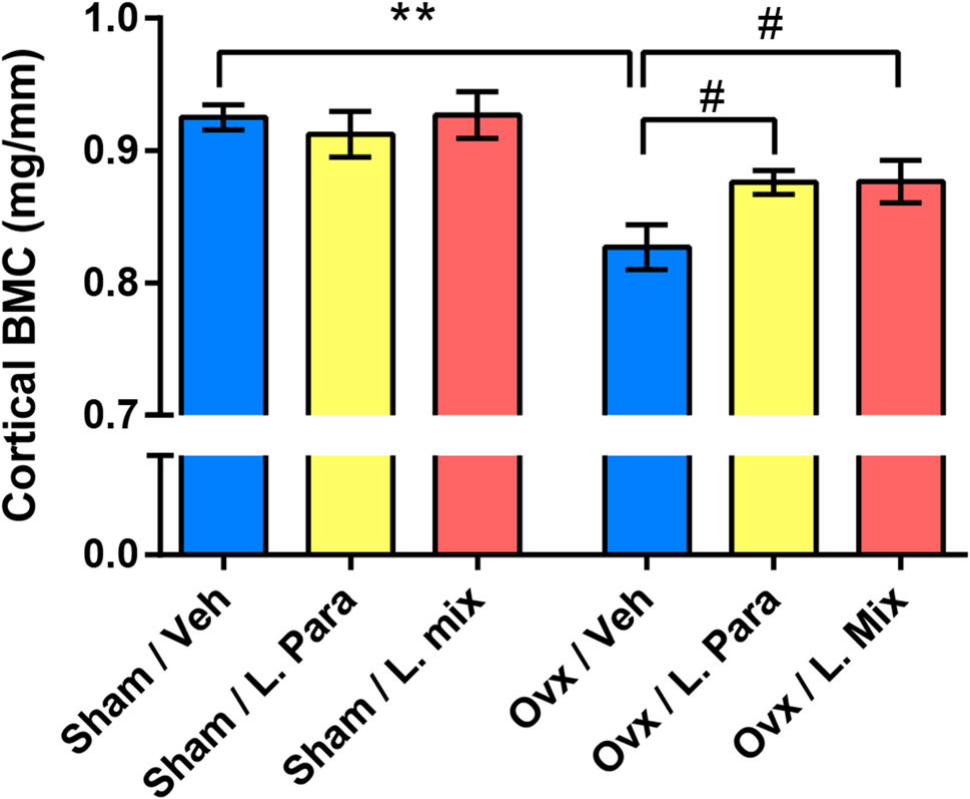
Probiotics protect mice from ovariectomy (ovx) induced cortical bone loss. Eight-week-old mice were treated with either vehicle (veh), a single *Lactobacillus* (*L*) strain (*L. para*), or a mixture of three strains (*L. mix*) for 6 weeks, starting 2 weeks before ovx or sham surgery to study the preventive effect of probiotic treatment on ovx-induced bone loss. At the end of the experiment, the dissected femurs were analyzed with computed tomography. Cortical bone mineral content (BMC) was measured in the mid-diaphyseal region of femur. Values are given as mean ± SEM, **p ≤ 0.01, *p ≤ 0.05. Student’s *t* test ovx vs. sham.#*p* ≤ 0.05, ANOVA followed by Dunnett’s post hoc test within the groups, ovx L. Para and L. mix versus ovx veh. Figure and legend reproduced from Ohlsson et al. [41] with permission from PLOS

As several studies indicate that alteration of the GM by antibiotic treatment modulates the magnitude of bone loss in sex steroid-deficient female mice, we hypothesized that treatment with probiotics might protect mice from ovariectomy (ovx)-induced bone loss [38, 39]. Based on their anti-inflammatory properties, we selected a single [*L. paracasei* DSM13434 (*L. para*)] and a mixture [*L. paracasei* DSM13434, *L. plantarum* DSM 15312 and DSM 15313 (*L. mix*)] of probiotic strains [40], and these were given in the drinking to ovx mice starting 2 weeks before ovx. Both the *L. para* and the *L. mix* treatments protected the mice from ovx-induced bone loss (Fig. 3) [41]. Probiotic treatment reduced the expression of two inflammatory cytokines, TNFα and IL-1β, and increased the expression of OPG, a potent inhibitor of osteoclastogenesis, in bone of ovx mice. A bone protective effect of probiotic treatment has also been reported by Britton R. A. et al. They tested the effect of *Lactobacillus reuteri* (*L. reuteri*), a commensal bacterium that also secretes beneficial immunomodulatory factors, on ovx-induced bone loss. Treatment with *L. reuteri* altered the GM composition and prevented ovx-induced trabecular bone loss and bone resorption [42]. In addition, this treatment suppressed ovx-induced increase in bone marrow CD4+T cells, supporting the notion that the GM modulates the immune status in bone and thereby affects osteoclast-mediated bone resorption. In a study by Parvaneh et al., the probiotic bacteria *Bifidobacterium longum* protected rats from ovx-induced bone loss [43]. It was recently demonstrated in an important study that GF mice are protected from trabecular bone loss induced by sex steroid depletion and that in CONV-R mice that lost bone, sex steroid deficiency increased gut permeability and upregulated the levels of osteoclastogenic cytokines in the small intestine and the bone marrow [44]. These findings suggest that the effect of sex steroid deficiency on bone mass could be mediated by increased gut permeability and thereby increased antigen load passing through the intestinal barrier activating immune cells. It was also demonstrated that the bone loss and increased gut permeability induced by ovx could be prevented by probiotic treatment.

**Fig. 4:**
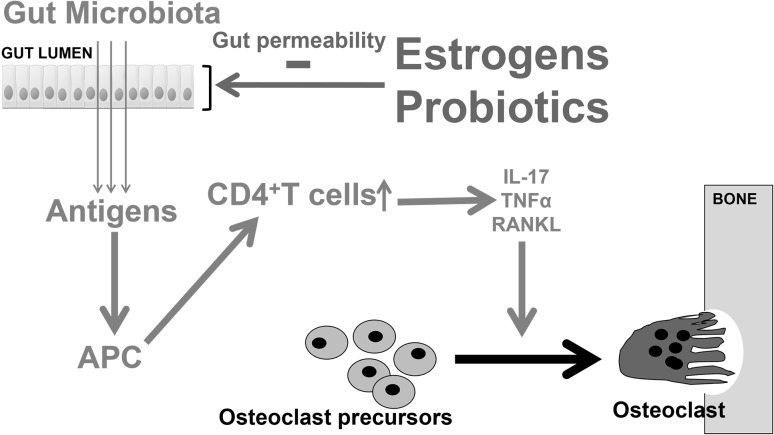
Proposed model for how estrogens and probiotics modulate the immune-mediated effect of GM on bone mass via a reduction of gut permeability. GM products act as antigens passing through the gap junctions between the intestinal cells and interact with antigen-presenting cells. This results in the activation of the immune system and specifically of CD4+T cells producing osteoclastogenic cytokines such as IL17, TNFα, and RANKL promoting osteoclastogenesis, bone resorption, and bone loss. This cascade is dependent on gut permeability and thereby the antigen load passing through the intestinal barrier. Li et al. showed that sex steroid depletion increases gut permeability and the production of osteoclastogenic cytokines in the bone marrow [29]. In addition, they showed that mice housed under GF conditions are protected against intestinal and bone marrow inflammatory responses and the loss of trabecular bone induced by sex steroid deficiency. Both estrogen and probiotic treatments could reduce GM permeability and thereby exert a bone protective effect in ovx mice. Thus, they proposed that one of the mechanisms for probiotics to protect from ovx-induced bone loss could be to reduce gut permeability and thereby counteract the effect of sex steroid deficiency

Based on this study, Li et al. proposed that GM products act as antigens passing through the intestinal cells and interact with antigen-presenting cells (Fig. 4). This results in the activation of the immune system including CD4+T cells producing osteoclastogenic cytokines promoting osteoclastogenesis, bone resorption, and bone loss. This cascade is dependent on the gut permeability and thereby the antigen load passing through the intestinal barrier. The gut permeability can be reduced by probiotics and estrogens, protecting the host from inflammation-induced bone loss (Fig. 4).

The first small (21 subjects in each group) short-term (6 months) placebo-controlled clinical study on the effects of a probiotic supplement on bone health in osteopenic postmenopausal women was recently published [45]. Consumption of the probiotic supplement containing seven probiotic bacterial species decreased bone turnover markers and the inflammatory cytokine TNFα in serum compared to the placebo group, but no significant effect on bone mineral density was observed. Future long-term studies in well-powered study populations will reveal if probiotic treatment has the capacity to influence bone mineral density in humans. According to Clinical Trials.gov (www.clinicaltrials.gov; Sept 2, 2017), there are currently three ongoing double-blind, placebo-controlled, randomized clinical studies of the effect of 12-month treatment with probiotics on bone mineral density in postmenopausal women or in osteopenic elderly women. The bacterial strains used in these three clinical studies were very recently all shown to protect mice from ovx-induced osteoporosis in three independent animal studies [29, 41, 42]. Thus, the translation from experimental animal studies to the initiation of clinical osteoporosis trials has been rapid in this field using probiotic treatments defined as dietary supplements.

## Summary and Future Perspectives

Animal studies demonstrate that the GM is a crucial regulator of bone metabolism and that specific modulation of the GM composition by probiotic interventions can prevent ovx-induced bone loss. The overall immune-related effect of short-term colonization of germ-free mice with GM is an activation of CD4+T cells, resulting in increased levels of pro-inflammatory cytokines in bone and thereby activation of osteoclastic bone resorption. Besides these immune-mediated effects on bone mass, the GM is involved in nutritional uptake and may, thereby, regulate overall body growth and bone sizes, possibly mediated via altered IGF-I levels.

We recently introduced a new term “osteomicrobiology” for the rapidly emerging research field of the role of the microbiota in bone health and disease. This research field is aimed to bridge the gaps between bone physiology, gastroenterology, immunology, and microbiology. There has been a rapid translation from experimental animal studies to ongoing clinical trials of the effect of certain probiotic treatments on bone health in postmenopausal women and the results from these studies will be informative to determine if the GM might be a novel therapeutic target for osteoporosis. In addition, several large-scale cohort studies are initiated to determine if the GM composition might be used as a biomarker for fracture prediction.

## References

[1] Sambrook P, Cooper C (2006). Osteoporosis. Lancet.

[2] Ralston SH, Uitterlinden AG (2010). Genetics of osteoporosis. Endocr Rev.

[3] Kearns AE, Khosla S, Kostenuik PJ (2008). Receptor activator of nuclear factor kappaB ligand and osteoprotegerin regulation of bone remodeling in health and disease. Endocr Rev.

[4] Lorenzo J, Horowitz M, Choi Y (2008). Osteoimmunology: interactions of the bone and immune system. Endocr Rev.

[5] D’Amelio P, Sassi F (2016). Osteoimmunology: from mice to humans. Bonekey Rep.

[6] Kong YY, Feige U, Sarosi I, Bolon B, Tafuri A, Morony S, Capparelli C, Li J, Elliott R, McCabe S, Wong T, Campagnuolo G, Moran E, Bogoch ER, Van G, Nguyen LT, Ohashi PS, Lacey DL, Fish E, Boyle WJ, Penninger JM (1999). Activated T cells regulate bone loss and joint destruction in adjuvant arthritis through osteoprotegerin ligand. Nature.

[7] Martin-Millan M, Almeida M, Ambrogini E, Han L, Zhao H, Weinstein RS, Jilka RL, O’Brien CA, Manolagas SC (2010). The estrogen receptor-alpha in osteoclasts mediates the protective effects of estrogens on cancellous but not cortical bone. Mol Endocrinol.

[8] Nakamura T, Imai Y, Matsumoto T, Sato S, Takeuchi K, Igarashi K, Harada Y, Azuma Y, Krust A, Yamamoto Y, Nishina H, Takeda S, Takayanagi H, Metzger D, Kanno J, Takaoka K, Martin TJ, Chambon P, Kato S (2007). Estrogen prevents bone loss via estrogen receptor alpha and induction of Fas ligand in osteoclasts. Cell.

[9] Pasco JA, Kotowicz MA, Henry MJ, Nicholson GC, Spilsbury HJ, Box JD, Schneider HG (2006). High-sensitivity C-reactive protein and fracture risk in elderly women. JAMA.

[10] Ding C, Parameswaran V, Udayan R, Burgess J, Jones G (2008). Circulating levels of inflammatory markers predict change in bone mineral density and resorption in older adults: a longitudinal study. J Clin Endocrinol Metab.

[11] Schett G, Kiechl S, Weger S, Pederiva A, Mayr A, Petrangeli M, Oberhollenzer F, Lorenzini R, Redlich K, Axmann R, Zwerina J, Willeit J (2006). High-sensitivity C-reactive protein and risk of nontraumatic fractures in the Bruneck study. Arch Intern Med.

[12] Eriksson AL, Moverare-Skrtic S, Ljunggren O, Karlsson M, Mellstrom D, Ohlsson C (2014). High-sensitivity CRP is an independent risk factor for all fractures and vertebral fractures in elderly men: the MrOS Sweden study. J Bone Miner Res.

[13] Charatcharoenwitthaya N, Khosla S, Atkinson EJ, McCready LK, Riggs BL (2007). Effect of blockade of TNF-alpha and interleukin-1 action on bone resorption in early postmenopausal women. J Bone Miner Res.

[14] Qin J, Li R, Raes J, Arumugam M, Burgdorf KS, Manichanh C, Nielsen T, Pons N, Levenez F, Yamada T, Mende DR, Li J, Xu J, Li S, Li D, Cao J, Wang B, Liang H, Zheng H, Xie Y, Tap J, Lepage P, Bertalan M, Batto JM, Hansen T, Le Paslier D, Linneberg A, Nielsen HB, Pelletier E, Renault P, Sicheritz-Ponten T, Turner K, Zhu H, Yu C, Jian M, Zhou Y, Li Y, Zhang X, Qin N, Yang H, Wang J, Brunak S, Dore J, Guarner F, Kristiansen K, Pedersen O, Parkhill J, Weissenbach J, Bork P, Ehrlich SD (2010). A human gut microbial gene catalogue established by metagenomic sequencing. Nature.

[15] Antonopoulos DA, Huse SM, Morrison HG, Schmidt TM, Sogin ML, Young VB (2009). Reproducible community dynamics of the gastrointestinal microbiota following antibiotic perturbation. Infect Immun.

[16] Dethlefsen L, Relman DA (2011). Incomplete recovery and individualized responses of the human distal gut microbiota to repeated antibiotic perturbation. Proc Natl Acad Sci U S A.

[17] Kau AL, Ahern PP, Griffin NW, Goodman AL, Gordon JI (2011). Human nutrition, the gut microbiome and the immune system. Nature.

[18] Schroeder BO, Backhed F (2016). Signals from the gut microbiota to distant organs in physiology and disease. Nat Med.

[19] Blumberg R, Powrie F (2012). Microbiota, disease, and back to health: a metastable journey. Sci Transl Med.

[20] Tremaroli V, Backhed F (2012). Functional interactions between the gut microbiota and host metabolism. Nature.

[21] Maynard CL, Elson CO, Hatton RD, Weaver CT (2012). Reciprocal interactions of the intestinal microbiota and immune system. Nature.

[22] Schmidt S, Mellstrom D, Norjavaara E, Sundh V, Saalman R (2012). Longitudinal assessment of bone mineral density in children and adolescents with inflammatory bowel disease. J Pediatr Gastroenterol Nutr.

[23] Stotzer PO, Johansson C, Mellstrom D, Lindstedt G, Kilander AF (2003). Bone mineral density in patients with small intestinal bacterial overgrowth. Hepatogastroenterology.

[24] Macpherson AJ, Harris NL (2004). Interactions between commensal intestinal bacteria and the immune system. Nat Rev Immunol.

[25] Faith JJ, Ahern PP, Ridaura VK, Cheng J, Gordon JI (2014). Identifying gut microbe-host phenotype relationships using combinatorial communities in gnotobiotic mice. Sci Transl Med.

[26] Sjogren K, Engdahl C, Henning P, Lerner UH, Tremaroli V, Lagerquist MK, Backhed F, Ohlsson C (2012). The gut microbiota regulates bone mass in mice. J Bone Miner Res.

[27] Yadav VK, Ryu JH, Suda N, Tanaka KF, Gingrich JA, Schutz G, Glorieux FH, Chiang CY, Zajac JD, Insogna KL, Mann JJ, Hen R, Ducy P, Karsenty G (2008). Lrp5 controls bone formation by inhibiting serotonin synthesis in the duodenum. Cell.

[28] Cui Y, Niziolek PJ, MacDonald BT, Zylstra CR, Alenina N, Robinson DR, Zhong Z, Matthes S, Jacobsen CM, Conlon RA, Brommage R, Liu Q, Mseeh F, Powell DR, Yang QM, Zambrowicz B, Gerrits H, Gossen JA, He X, Bader M, Williams BO, Warman ML, Robling AG (2011). Lrp5 functions in bone to regulate bone mass. Nat Med.

[29] Li JY, Chassaing B, Tyagi AM, Vaccaro C, Luo T, Adams J, Darby TM, Weitzmann MN, Mulle JG, Gewirtz AT, Jones RM, Pacifici R (2016). Sex steroid deficiency-associated bone loss is microbiota dependent and prevented by probiotics. J Clin Invest.

[30] Ohlsson C, Nigro G, Boneca IG, Backhed F, Sansonetti P, Sjogren K (2017). Regulation of bone mass by the gut microbiota is dependent on NOD1 and NOD2 signaling. Cell Immunol.

[31] Novince CM, Whittow CR, Aartun JD, Hathaway JD, Poulides N, Chavez MB, Steinkamp HM, Kirkwood KA, Huang E, Westwater C, Kirkwood KL (2017). Commensal gut microbiota immunomodulatory actions in bone marrow and liver have catabolic effects on skeletal homeostasis in health. Sci Rep.

[32] Park OJ, Kim J, Yang J, Yun CH, Han SH (2017). Muramyl dipeptide, a shared structural motif of peptidoglycans, is a novel inducer of bone formation through induction of runx2. J Bone Miner Res.

[33] Fazeli PK, Klibanski A (2014). Determinants of GH resistance in malnutrition. J Endocrinol.

[34] Schwarzer M, Makki K, Storelli G, Machuca-Gayet I, Srutkova D, Hermanova P, Martino ME, Balmand S, Hudcovic T, Heddi A, Rieusset J, Kozakova H, Vidal H, Leulier F (2016). Lactobacillus plantarum strain maintains growth of infant mice during chronic undernutrition. Science.

[35] Yan J, Herzog JW, Tsang K, Brennan CA, Bower MA, Garrett WS, Sartor BR, Aliprantis AO, Charles JF (2016). Gut microbiota induce IGF-1 and promote bone formation and growth. Proc Natl Acad Sci U S A.

[36] Li JY, Chassaing B, Tyagi AM, Vaccaro C, Luo T, Adams J, Darby TM, Weitzmann MN, Mulle JG, Gewirtz AT, Jones RM, Pacifici R (2016). Sex steroid deficiency-associated bone loss is microbiota dependent and prevented by probiotics. J Clin Invest.

[37] Sanders ME, Guarner F, Guerrant R, Holt PR, Quigley EM, Sartor RB, Sherman PM, Mayer EA (2013). An update on the use and investigation of probiotics in health and disease. Gut.

[38] Williams S, Wakisaka A, Zeng QQ, Barnes J, Martin G, Wechter WJ, Liang CT (1996). Minocycline prevents the decrease in bone mineral density and trabecular bone in ovariectomized aged rats. Bone.

[39] Pytlik M, Folwarczna J, Janiec W (2004). Effects of doxycycline on mechanical properties of bones in rats with ovariectomy-induced osteopenia. Calcif Tissue Int.

[40] Lavasani S, Dzhambazov B, Nouri M, Fak F, Buske S, Molin G, Thorlacius H, Alenfall J, Jeppsson B, Westrom B (2010). A novel probiotic mixture exerts a therapeutic effect on experimental autoimmune encephalomyelitis mediated by IL-10 producing regulatory T cells. PLoS ONE.

[41] Ohlsson C, Engdahl C, Fak F, Andersson A, Windahl SH, Farman HH, Moverare-Skrtic S, Islander U, Sjogren K (2014). Probiotics protect mice from ovariectomy-induced cortical bone loss. PLoS ONE.

[42] Britton RA, Irwin R, Quach D, Schaefer L, Zhang J, Lee T, Parameswaran N, McCabe LR (2014). Probiotic L. reuteri treatment prevents bone loss in a menopausal ovariectomized mouse model. J Cell Physiol.

[43] Parvaneh K, Ebrahimi M, Sabran MR, Karimi G, Hwei AN, Abdul-Majeed S, Ahmad Z, Ibrahim Z, Jamaluddin R (2015). Probiotics (Bifidobacterium longum) increase bone mass density and upregulate sparc and bmp-2 genes in rats with bone loss resulting from ovariectomy. Biomed Res Int.

[44] Li J-Y, Reott M, Adams J, Weitzmann MN, Gewirtz A, Pacifici R (2014). Gut microbiota plays a pivotal role in the bone loss induced by sex steroid deficiency. J Bone Miner Res.

[45] Jafarnejad S, Djafarian K, Fazeli MR, Yekaninejad MS, Rostamian A, Keshavarz SA (2017). Effects of a multispecies probiotic supplement on bone health in osteopenic postmenopausal women: a randomized, double-blind. Control Trial J Am Coll Nutr.

